# Genome-wide identification of *heavy-metal ATPases* genes in *Areca catechu*: investigating their functionality under heavy metal exposure

**DOI:** 10.1186/s12870-024-05201-6

**Published:** 2024-05-31

**Authors:** Noor Muhammad Khan, Akhtar Ali, Yinglang Wan, Guangzhen Zhou

**Affiliations:** 1 Hainan Key Laboratory for Sustainable Utilization of Tropical Bioresources, School of Tropical Agriculture and Forestry, Hainan University, Haikou, China; 2 Ministry of Education Key Laboratory for Ecology of Tropical Islands, Key Laboratory of Tropical Animal and Plant Ecology of Hainan Province, College of Life Sciences, Hainan Normal University, Haikou, Hainan China

**Keywords:** *Areca catechu*, *HMA* gene family, Gene expression profile, Heavy metal, Abiotic stress

## Abstract

Heavy-metal ATPases (HMAs) play a vital role in plants, helping to transport heavy metal ions across cell membranes.However, insufficient data exists concerning HMAs genes within the *Arecaceae* family.In this study, 12 *AcHMA* genes were identified within the genome of *Areca catechu*, grouped into two main clusters based on their phylogenetic relationships.Genomic distribution analysis reveals that the *AcHMA* genes were unevenly distributed across six chromosomes. We further analyzed their physicochemical properties, collinearity, and gene structure.Furthermore, RNA-seq data analysis exhibited varied expressions in different tissues of *A. catechu* and found that *AcHMA1*, *AcHMA2*, and *AcHMA7* were highly expressed in roots, leaves, pericarp, and male/female flowers. A total of six *AcHMA* candidate genes were selected based on gene expression patterns, and their expression in the roots and leaves was determined using RT-qPCR under heavy metal stress. Results showed that the expression levels of *AcHMA1* and *AcHMA3* genes were significantly up-regulated under Cd2 + and Zn2 + stress. Similarly, in response to Cu^2+,^ the *AcHMA5* and *AcHMA8* revealed the highest expression in roots and leaves, respectively. In conclusion, this study will offer a foundation for exploring the role of the *HMAs* gene family in dealing with heavy metal stress conditions in *A. catechu*.

**Supplementary Information:**

The online version contains supplementary material available at 10.1186/s12870-024-05201-6.

## Introduction

Heavy metal toxicity poses severe threats to crop yields worldwide [1]. Plants are sensitive to metal toxicity because it impacts their growth and productivity, reduces their water use efficiency, and causes various physiological and biochemical changes in plants [2]. Heavy metal contamination of soil, water, and food poses a significant health risk to the human body [3]. Plants need heavy metals such as copper, manganese, zinc, nickel, and cobalt for various biological processes [4], whereas some other non-essential toxic metals, such as lead (Pb) and cadmium (Cd), can be absorbed by plants, which are highly toxic and adversely affect crop productivity [5]. Several metabolic pathways in plants are inhibited by high concentrations of Cd^2+^ and Cu^2+^, including but not limited to photosynthesis, respiration, and enzyme activity. These ions decrease protein and chlorophyll levels, increase ROS production, and alter gene expression associated with oxidative stress responses [6, 7]. Zinc (Zn) and Iron (Fe) are essential elements involved in photosynthesis and cell respiration. Zinc is a cofactor and structural element of various transcription factors and structural proteins [8]. However, a high Zn^2+^ concentration can affect plant growth, chlorosis, necrosis in leaves, browning of roots, and the degradation of cell membrane integrity and permeability [9]. Plants employ two main strategies to mitigate the adverse effects of heavy metals and sustain normal metabolic activities: limiting the absorption of heavy metals from the soil and accumulating them within vacuoles [10]. The defensive mechanisms of various metal ion transporters control the coordination and balancing of metal ions in plants. Numerous metal transporters have been identified, including cation diffusion facilitator proteins (CDF), Zn-regulated transporter-like proteins (ZNT/ZIP), yellow stripe proteins (YSL), and ATP-binding cassette proteins (ABC). Natural resistance-associated macrophage proteins (NRAMP), Metal tolerance proteins (MTP), and heavy metal ATPases (HMA) are actively involved in metal transport. These transporters collectively contribute to effectively regulating plant metal ion levels, highlighting the complexity and cooperation in maintaining metal homeostasis [11–13].

Heavy-metal ATPase (HMA), or P1B-type ATPase, is one of the most important transporters of heavy metal ions. It combines ATP hydrolysis with metal ions to facilitate the absorption and translocation of heavy metal ions (such as Zn^2+^, Cd^2+^, Cu^2+^, Co^2+^, and Pb^2+^) across cell membranes [14, 15]. A typical HMAs possess 6 to 8 transmembrane helices (TMs), an H.P. locus, and a CPx/SPC motif essential for metal transport and metal binding domains at both C and N-terminal regions [16]. Based on their specific metal substrate binding properties, ATPases can be classified as Cu/Ag-ATPases or Zn/Co/Cd/Pb-ATPases [17–19]. At present, the HMA proteins have been characterized in many plants, i.e., *A. thaliana*, maize (*Zea mays L*.), rice *(Oryza sativa)*, and wheat *(T. aestivum)* etc., at the genomic and molecular level [14, 20–22]. The functionality of HMA genes varies across different plant species, each contributing to specific roles. The heterologous expression of *AtHMA1* in *Saccharomyces cerevisiae* shows its role in detoxifying excessive Zn (II) in the chloroplast [23]. The *OsHMA2*, *AtHMA2*, and *AtHMA4* perform a crucial role in Zn^2+^ and Cd^2+^ translocation from the xylem of the roots to the shoots of the plant [24, 25]. Overexpression of *AtHMA3* increases 2- to 3-fold tolerance against heavy metals and accumulates Cd, Zn, Pb, and Co to the vacuoles [26]. In *O. sativa*, *OsHMA1*, and *OsHMA2* are supposed to be involved in Zn transportation in tonoplast [14, 15, 27]. *OsHMA3* acts as a Cd, whereas *OsHMA4* transports Cu to the roots cell vacuoles [28–30].

*Areca catechu L*., is a versatile plant in the palm family, holds significant medicinal and economic value, and is grown across China, India, Thailand, Indonesia, Malaysia, and Cambodia [31, 32]. The areca nut, characterized by its abundant arecoline content and regarded as the fourth most addictive globally, following alcohol, caffeine, and nicotine, has emerged as a crucial agricultural commodity in Southeast Asia and Africa. Although the complete genome sequence of *Areca* has been published, there remains a gap in the literature regarding a comprehensive examination of genome-wide analysis and expression profiling of the *AcHMA* family. In this study, we performed a genome-wide analysis of *AcHMA* gene family in *A. catechu*. A total of 12 *AcHMA* genes were identified, and they were analyzed for their chromosomal locations, phylogenies, conserved motifs, structures, and expression profiles, as well as their responses to heavy metals (CuSO_4_, ZnSO_4_, and CdCl_2_). Additionally, the study of *AcHMA* will be of guiding significance for understanding the transport of heavy metal ions in palm plants.

## Materials and methods

### *Retrieval of the AcHMA gene family in A. catechu*

The *A. catechu* (Taxonomy ID: 184,783) whole-genome assembly protein sequences were obtained from NCBI (ID: JAHSVC000000000; BioSample: SAMN19591864; Accession: PRJNA735650). The conserved HMA protein domain (PF00403), E1-E2_ATPase domain (PF00122), and hydrolase domain (PF00702) were retrieved from the Pfam database (http://pfam.sanger.ac.uk/search (accessed on November 15, 2023). The *HMA* gene family in *A. catechu* was identified using two methods. In the first method, the *HMAs* (*AtHMA1-AtHMA8*) protein sequences of *Arabidopsis* were retrieved from TAIR (http://www.arabidopsis.org accessed on November 15, 2023) to be used as query proteins. Using BioEdit v7.2.6, a local protein sequence database of *A.catechu* was created. BLASTp was used to identify predictable HMA genes in *A. catechu* using the *Arabidopsis* genome.

In the second approach, we performed a Hidden Markov Model (HMM) search against the genome of *A. catechu* to conform the presence of HMA genes using HMMER version 3.2.1 with default settings (http://hmmer.org/ (accessed on November 15, 2023)). After merging the results, the HMA genes were further screened on the basis of their domain composition.

Subsequently, any missing conserved and duplicated sequences identified by the two methods were manually eliminated. The merged findings were further screened based on their domain composition in the NCBI Conserved Domains Database (https://www.ncbi.nlm.nih.gov/Structure/cdd/cdd.shtml (accessed on November 15, 2023)) and the SMART database (http://smart.embl-heidelberg.de/ (accessed on November 15, 2023)). Protein sequences without the conserved HMA Pfam domains, errors or shorter (< 100aa) were removed and those with an E-value < 1 *×* 10^*−* 10^ were retained.

### Analysis of physicochemical properties of *AcHMAs*

A variety of physiochemical properties of HMAs were identified by using the ExPASy server (http://web.expasy.org/protparam/ (accessed on November 15, 2023)), including amino acid length, predicted molecular weight, Gravy, and theoretical isoelectric points. The protein subcellular localization Prediction Tool (PSORT) (https://www.genscript.com/psort.html) was used for the subcellular localization prediction of all *AcHMA* genes.

### Phylogenetic, gene synteny analysis and Ka/Ks calculation

The HMAs protein sequences of *Arabidopsis* were downloaded from TAIR (http://www.arabidopsis.org), *O. sativa* from Phytozome (https://phytozome.jgi.doe.gov), and *Cocos nucifera* from ((https://arecaceae-gdb.com/#/) (accessed on November 20, 2023). ClustalW was used for multiple sequence alignment of all identified *AcHMA* gene family proteins. MEGA 11.0 software generated the phylogenetic tree based on the Maximum Likelihood method with default parameters: bootstrapping to 1000, Poisson model, and complete deletion (accessed November 20, 2023). The alignment sequences were trimmed with TBtools software [33]. Evolgenius (https://www.evolgenius.info/evolview-v2/#mytrees/ia-tree6.0/6.0) was used to visualize and optimize the tree. MCScanX was set with its default parameters to identify gene duplication events and syntenic relationships. The outcomes were then graphically represented using Circos and a Dual Synteny Plot generated with TBtools. The non-synonymous and the synonymous substitution ratio (Ka/Ks) of homologous *HMA* gene pairs in *A. thaliana, A. catechu, O. sativa, and C. nucifera* were calculated using TBtools.

According to the Ka/Ks ratio, duplicate genes are detected under positive or negative selection (if the ratio is > 1, neutral selection is suggested, and Ka/Ks ratios of less than one offer negative or purifying selection) [34]. The divergence time of gene pairs was estimated using the synonymous mutation rate per synonymous site per year formula: T = Ks / 2x, where x = 6.56^10^− 9^ (He et al., 2016).

### Gene structure analysis, chromosomal distribution, and conserved motifs

Gene locations, chromosome lengths, exon/intron distributions were retrieved from the gene annotation file (GFF3) of the *A. catechu* genome and visualized via TBtools. Using the MEME online tool (http://meme-suite.org/tools/meme, accessed on November 15, 2023), the conserved motifs of *AcHMA* proteins were predicted and analyzed.

### Cis-acting elements and transmembrane topology

The promoter sequences, obtained from the *A. catechu* genome, were defined as the 2,000 bases upstream from the initial ATG of the coding sequence (CDS) of *AcHMA* genes. PlantCARE (http://bioinformatics.psb.ugent.be/webtools/plantcare/html/, accessed on November 15, 2023) was used to analyze the cis-element distribution in promoter regions.

### RNA-seq data analysis

The Illumina Hiseq 4000 RNA-seq data deposited at NCBI (accession number: PRJNA767949) were utilized for expression profiling analysis of *AcHMA* genes. FPKM values were computed for *AcHMA* genes based on RNA-seq data. Genes with a log2FC greater than 1, a false discovery rate of 0.05, and differentially expressed genes (DEGs) were determined based on a significance level with a p-value of less than 0.05. TBtools were utilized to plot the expression levels of *AcHMA* genes. Gene expression analyses were performed using BMK Cloud (www.biocloud.net).

### Plant materials and treatments

This study obtained *A. catechu* seedlings (Reyan No. 1) from the Coconut Research Institute of the Chinese Academy of Tropical Agricultural Sciences, Wenchang, Hainan province, China. The plants were cultivated at 28℃/25℃ (14/10 h day/night) photoperiod in pots (12 cm × 12 cm) with perlite at Hainan University, Hainan province, China. The plantlets were divided into two groups: Control (CK) and Treatment (T), after being treated for two weeks with half-strength Hoagland’s solution (pH = 6). The plantlets were treated with 100 µmol/L CdCl_2_, 100 µmol/L CuSO_4_,100 µM ZnSO_4_ [34] for 72 h to assess the expression patterns in comparison to the control group. Samples from roots and the top leaf were collected (*n* = 3) at four different periods (0, 12, 24, and 72 h) for each treatment, followed by quickly cryopreserved in liquid nitrogen and kept at -80 °C until RNA extraction.

### RNA isolation and qRT-PCR

RNA isolation was conducted using the plant RNA extraction kit (Code No. DP437, TIANGEN, Beijing, China). NanoDrop 2000 (KAIAO, Beijing, China) was used to detect RNA concentration and quality. TIANScript RT Kit (Code No. KR104, TIANGEN, Beijing, China) was used to generate first-strand cDNA. The reaction mixture containing 10 µL of vazyme Master Mix (Code No. Q111-02, Vazyme, Nanjing, China), 1 µL of forward and reverse primers, 1 µL cDNA template, and 7 µL ddH_2_O. Primer Premier 6.0 was used to design primer pairs, while actin was used as a control primer (Table S1). Each experiment consisted of three biological and technical replicates. The 2^−ΔΔCT^ method was employed to calculate the relative gene expression.

### Statistical analysis

The mean expression value was calculated using technical replicates. A Two-way ANOVA was conducted using Statistix 8.1, followed by the Tukey LSD test, to evaluate the presence of significant differences (*P* ≥ 0.05). The data was analyzed and histograms were generated using the Origin software.

## Results

### In silico characterization of HMA family in *A. catechu*

This study identified and characterized 12 *AcHMA* genes from the entire genome of A. *catechu*. The genes were assigned as *AcHMA1–AcHMA12* based on phylogenetic relations with known *A. thaliana HMAs* genes (Table S2). The total number of *HMA* family genes in the *A. catechu* were higher than that in the *A. thaliana* (8), rice (9), and maize (11) but lower than in soybean (20) *Brassica napus* (31). The physical and chemical properties revealed that the amino acid lengths of *AcHMA* proteins ranged from 428 amino acids (*AcHMA2*) up to 1030 (*AcHMA9*), whereas the average number was 812.5 amino acids. The M.W ranged from 45.76 kDa (*AcHMA2*) to 111.53 (*AcHMA9*), averaging 87.39 kDa. The theoretical isoelectric point (pI) varies from 5.41 (*AcHMA8*) to 8.09 (*AcHMA6*), with an average value of 6.68. The theoretical isoelectric point (pI) changed from 5.41 (*AcHMA8*) to 8.09 (*AcHMA6*) with an average value of 6.68, whereas the GRAVY values varied from 0.044 to 0.553 with an average range of 0.19. All genes had positive GRAVY values, indicating hydrophobic properties. The predicted subcellular localization reveals that most of the *AcHAMs* were located in the plasma membrane except two of them (*AcHMA1* and *AcHMA6*) were located in cytoplasm, and only one (*AcHMA12*) is found in the chloroplast (Table 1).

**Table 1:** Details of the *HMA* genes identified in *A. catechu* and their sequence characteristics

Gene ID	Gene name	Chr.	Genomic location	Protein length	M.W (kDa)	pI	GRAVY	Subcellular localization
AC10G097870	*AcHMA1*	10	159,824,328–159,851,926	638	68.16419	7.89	0.181	Chloroplast envelop
AC12G032050	*AcHMA2*	12	40,187,211–40,193,103	428	45.75887	6.26	0.553	Plasma Membrane
AC06G001100	*AcHMA3*	6	1,482,504–1,490,777	922	99.96247	6.56	0.044	Vacule envelop
AC10G001250	*AcHMA4*	10	1,298,536–1,332,906	924	100.5282	7.3	0.044	Plasma Membrane
AC08G025290	*AcHMA5*	8	31,755,667–31,764,520	975	105.29798	5.96	0.174	Plasma Membrane
AC04G017130	*AcHMA6*	4	24,311,115–24,342,499	959	101.2378	8.09	0.136	Plastid envelope
AC03G045730	*AcHMA7*	3	65,146,018–65,156,257	933	100.01856	5.45	0.284	Plasma Membrane
AC04G006760	*AcHMA8*	4	8,777,900–8,794,577	535	56.94047	5.41	0.226	Thylakoid Membrane
AC12G044360	*AcHMA9*	12	57,153,003–57,167,016	1030	111.53139	7.33	0.119	Plasma Membrane
AC12G044420	*AcHMA10*	12	57,286,658–57,297,633	890	96.56946	6.64	0.193	Plasma Membrane
AC12G044390	*AcHMA11*	12	57,222,464–57,237,801	995	107.30245	6.98	0.134	Plasma Membrane
AC12G047430	*AcHMA12*	12	61,726,077–61,738,374	521	55.41828	6.33	0.182	Plasma Membrane

### Phylogenetic and comparative analysis of HMAs in *A. catechu*

**Fig. 1:**
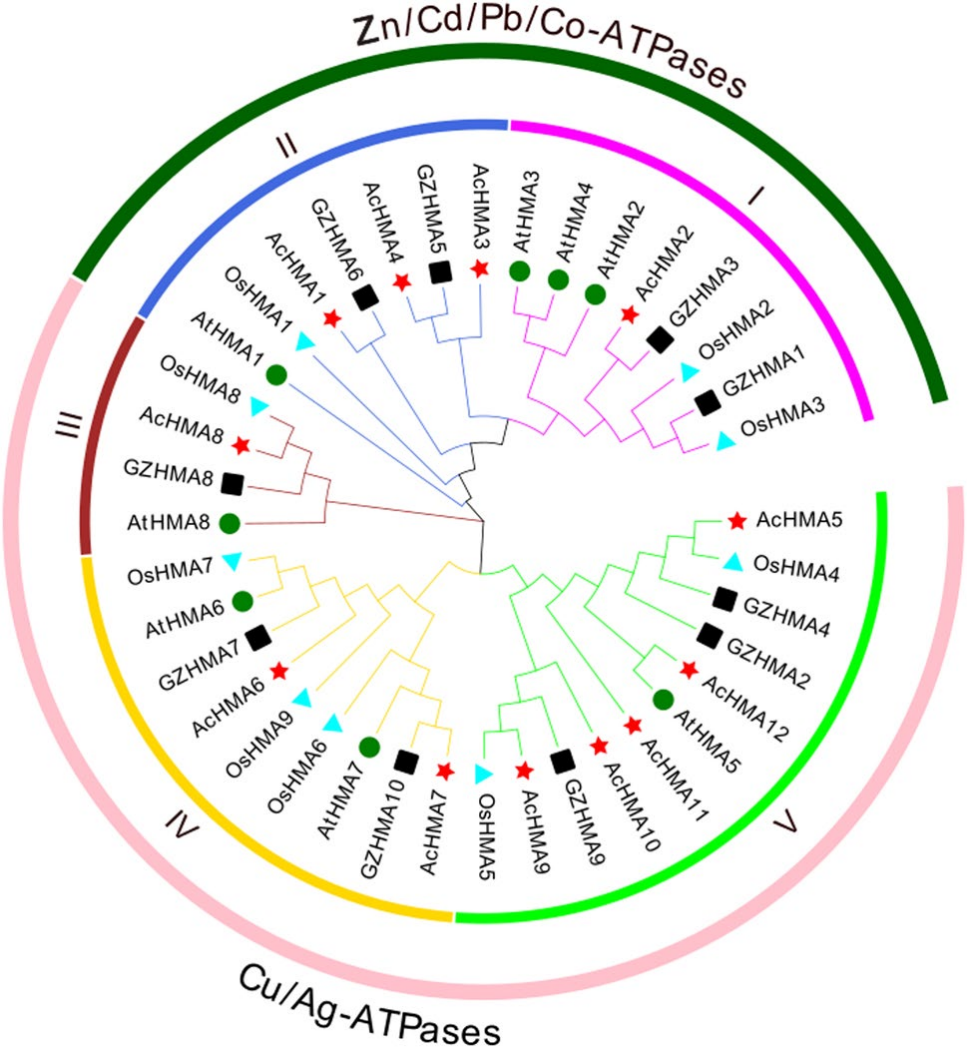
Phylogenetic relationship of HMA genes among *A. catechu*, *A. thaliana* and *O. sativa*. The tree was generated using MEGA11.0 based on the Maximum Likelihood (ML) of 39 HMA protein sequences. The tree were distributed into two main clades. These clades were further classified into Zn/Cd/Co-ATPases sub-groups (I and II) and the Cu/Ag-ATPases groups (III, IV, and V). Groups and subgroups are indicated by different background colors (green represents Zn/Cd/Co-ATPases; light pink represents Cu/Ag-ATPases subgroup). The red stars, green solid circles, and light blue triangles represent the *A. catechu*, *Arabidopsis*, and *O. sativa* HMA proteins

In order to investigate the evolutionary relations, we used 12 *HMA* genes from *A. catechu*, eight from *A. thaliana*, ten from *C. nucifera*, and nine from *O. sativa* to make a phylogenetic tree using the maximum likelihood method by MEGA11. Based on substrate specificity, 39 *HMA* genes were classified into two main groups (Cu/Ag-ATPases and Zn/Co/Cd/Pb-ATPases). The groups are further subdivided into five subgroups (I-V). *AcHMA* genes are distributed in all five subgroups, although group 5 has the most (5) genes. This discrepancy in orthologous pairs suggests a closer evolutionary relationship between the same species, *A. catechu*, and *C. nucifera*, than the more distant relationship with *A. thaliana* and *O. sativa*(Fig. 1).

### Chromosomal distribution, gene synteny analysis, and duplication

**Fig. 2:**
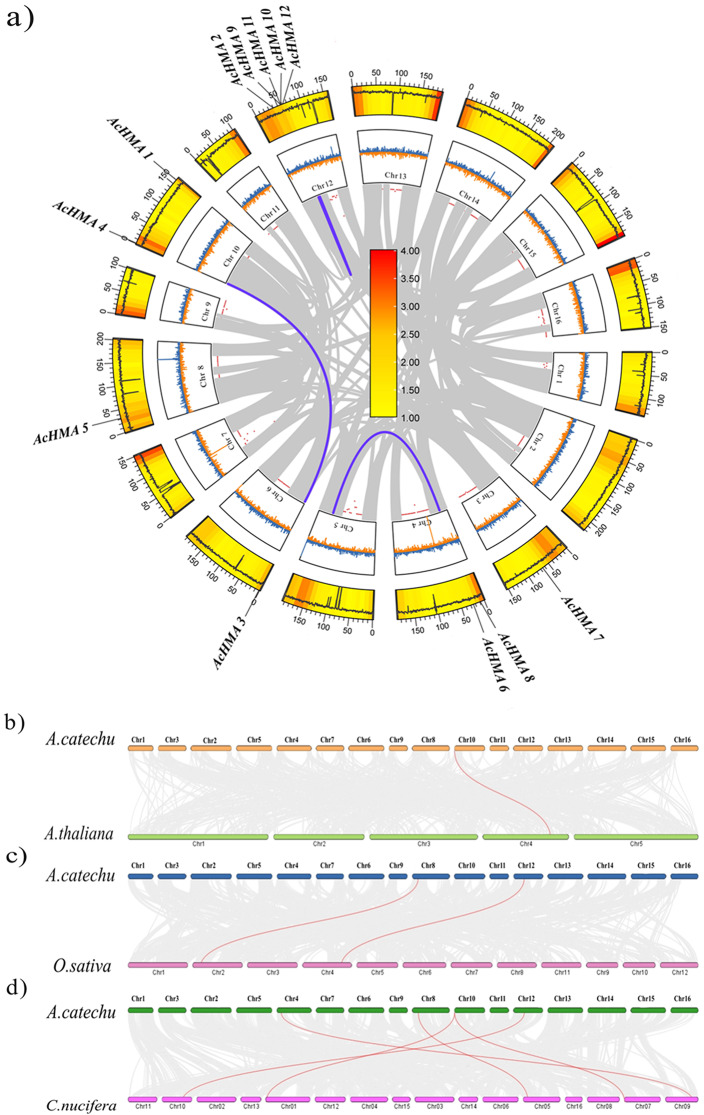
Synteny analysis of *AcHMAs*. (**a**)*AcHMAs* in the *A.catechu* genome and their colinear gene pairs. Chromosome numbers, gene density, and GC content are displayed from outer to inner rings. (**b**, **c**, **d**) Synteny analysis of *HMA* genes between areca and three representative plant species. Grey lines in the background indicate the collinear blocks within the orchardgrass and other plant genomes, whereas the red lines highlight the syntenic *HMA* gene pairs

The chromosomal location of 12 *AcHMAs* was analyzed to determine their genomic distribution. In total, twelve genes were found on six chromosomes, with chromosome twelve containing a maximum of five genes and chromosomes four and ten, each containing two genes (Fig. 2a). Synteny analysis was performed among family members of *AcHMAs* in *A.catechu* to investigate its possible evolutionary process. Three pairs of syntenic *AcHMA* genes (*AC04G017130.1*/*AC05G072680.1*, *AC10G001250.1*/*AC06G001050.1* and *AC12G044360.1*/ *AC12G047390.1*) were found in *A. catechu* (Fig. 2a). In order to gain a better understanding of the evolution mechanisms of the *AcHMA* gene family, syntenic analysis were constructed among *A.catechu* and three other species, including *A. thaliana* (dicot, *Brassicaceae*), *O. sativa* (monocot, *Poaceae*), and *C. nucifera* (*Arecaceae*). One *AcHMA* homologous gene pair was identified between *A. catechu* and *A. thaliana* (*AcHMA4*/ *AT4G30110.1*). Two homologous gene pairs were determined between *A. catechu* and *O. sativa (AcHMA5*/ *Os02g10290.1*; *AcHMA9*/ *Os04g46940.1*), and five *AcHMAs* homologous gene pairs were identified between *A. catechu* and *C. nucifera* (*AcHMA4*/*GZ01G0000730.1*; *AcHMA4*/*GZ07G0155070.1*; *AcHMA6*/ *GZ09G0200370.1*; *AcHMA5*/ *GZ05G0113160.1*; *AcHMA9*/ *GZ10G0210660.2*) (Fig. 2b-d; Table S3). To understand the divergence process of *AcHMA* homologous genes during replication, the Ka/Ks and its ratio were calculated within *A. catechu*, *A. thaliana*, *O. sativa*, and *C. nucifera* (Table S3). The Ka/Ks values were less than one (< 1), indicating purifying selection.

### Phylogenetic, conserved motifs and gene structure analysis

**Fig. 3:**
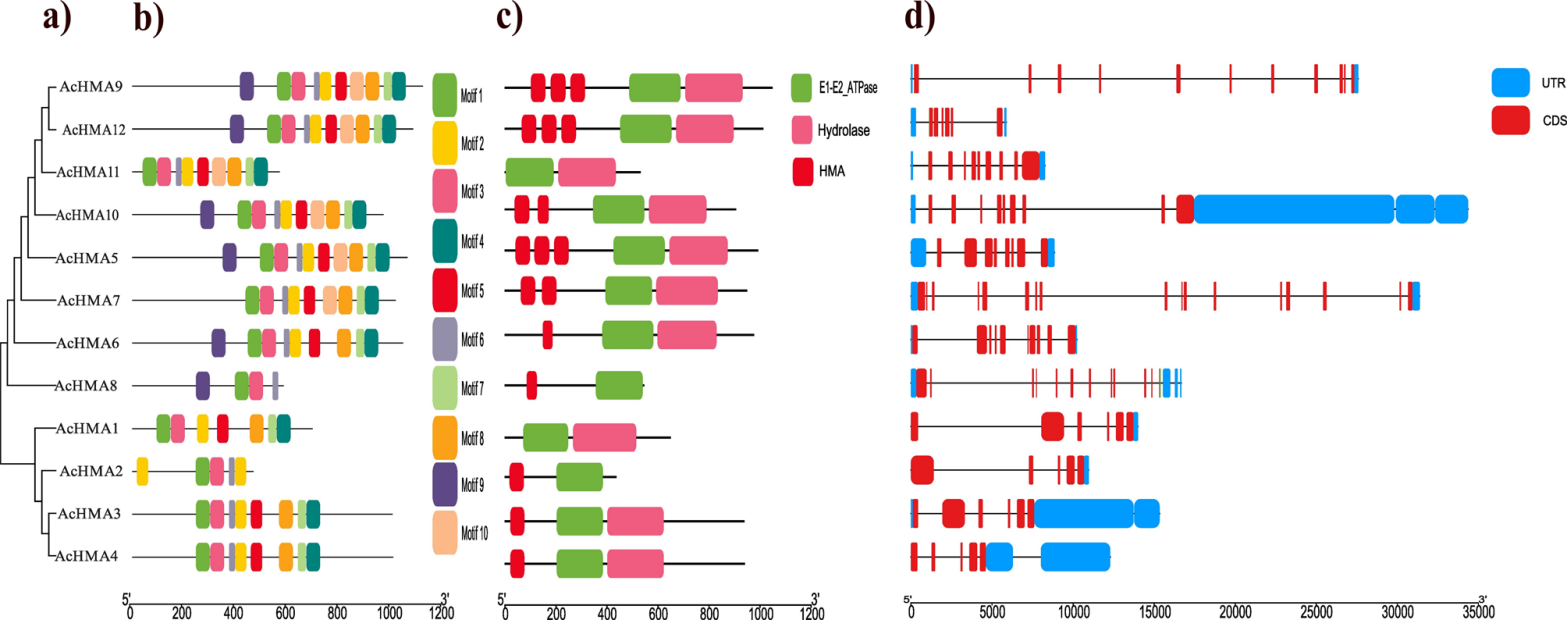
Phylogenetic analysis, conserved domains, exon-intron structure, and motif composition of *HMA*genes in *A. catechu* (**a**) Phylogenetic relationships of 12 *AcHMAs.* (**b**) MEME predicted the conserved motifs of HMA proteins. The different conserved motifs of HMA proteins are displayed as other colored boxes, and non-conserved sequences are shown as gray lines. (**c**) Distribution of conserved protein domains. (**d**) Schematic for *HMA* intron/exon structures. The gray lines show introns, the blue boxes indicate untranslated regions, and the red boxes represent CDS regions. The scale bar is shown at the bottom

The full-length AcHMA protein sequences were aligned to create an unrooted phylogenetic tree (Fig. 3a). The 12 AcHMAs were grouped into two main categories: Zn/Co/Cd/Pb-ATPases (AcHMA 1–4) and Cu/Ag-ATPases (AcHMA 5–12) based on their characteristics. The length of amino acid residues and the number of motifs were not conserved across the *AcHMA* group (Fig. 3b). Some motifs (motifs 1 and 3) were highly conserved in all group members, while a few (motifs 9 and 10) were only found in 6 group members. The Zn/Co/Cd/Pb subgroup, in comparison with Cu/Ag-ATPases, lacks several motif pairs. Detailed information about these motifs is shown in (Fig. 3b). Further, we also noticed that the position of the domains in the HMA proteins follows a similar pattern reported in *A. thaliana*. The Cu/Ag AcHMA proteins showed two types of domains, namely HMA and E1-E2-ATPase, while the Zn/Co/Cd/Pb AcHMA proteins exhibited mostly E1-E2-ATPase and hydrolase domains (Fig. 3c).

The intron-exon configurations of *AcHMA* genes is essential for unraveling their physiological roles. Through comparative analysis of the exon/intron ratio, it has been found that *AcHMA6* has the highest number of exons/intron (17/16) while *AcHMA10* has a minimum exons/intron (5/4) ratio across all *AcHMA* members (Table S4). In addition, the distribution of UTR numbers also varies from 1 to 5, where *AcHMA4* and *AcHMA8* have a maximum of 5 UTR while *AcHMA9* and *AcHMA10* have each 1 UTR, respectively (Fig. 3d; Table S4).The conserved motifs of AcHMA proteins were analyzed using the web tool MEME (www.meme-suiteorg/meme/tools/meme) to provide a more detailed understanding of their sequence characteristics (Table S5). The tool identified ten conserved protein motifs that exhibit significant conservation in terms of both their combination and relative position.

### Characterization of cis-acting elements within AcHMA promoter regions

**Fig. 4:**
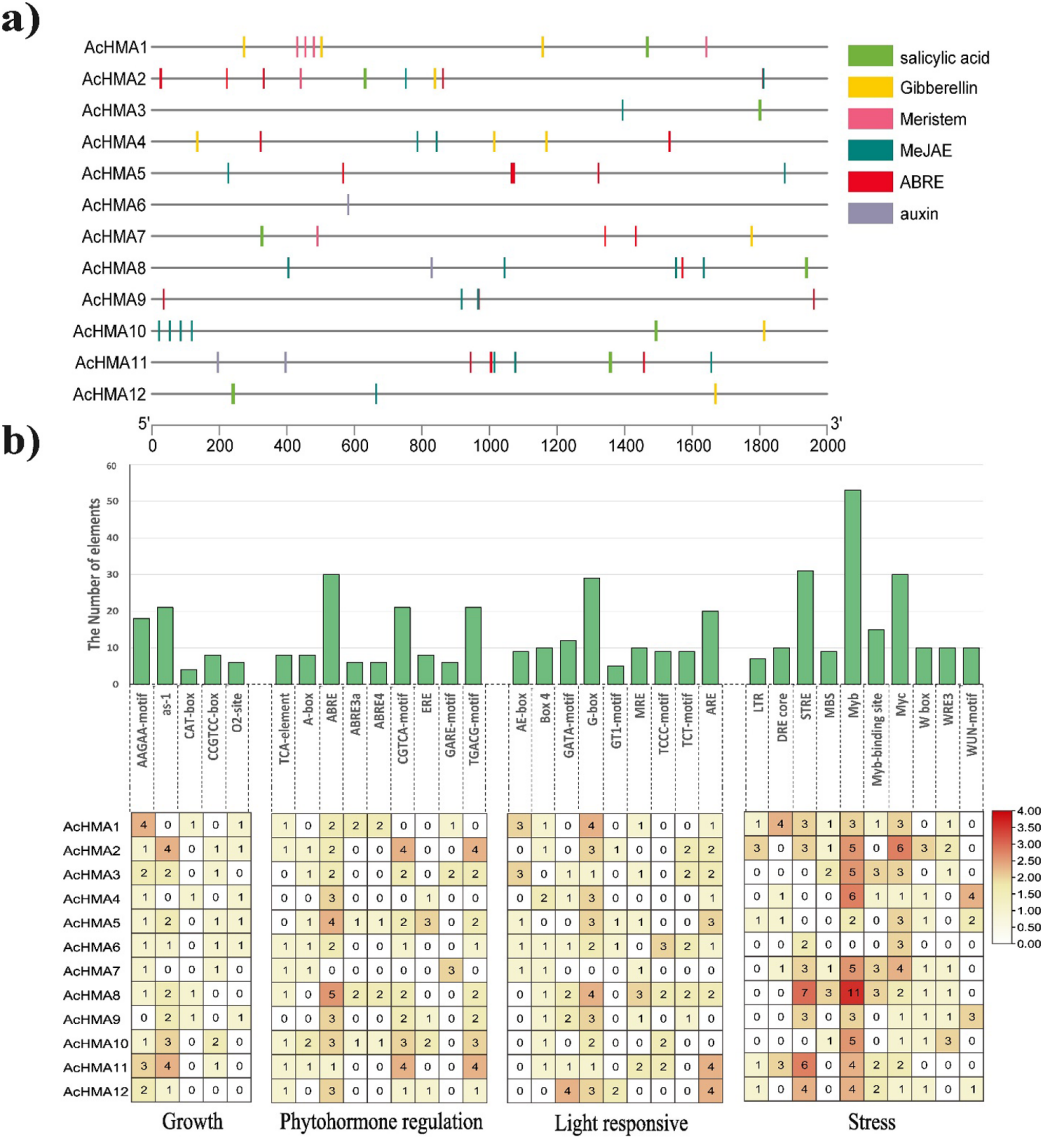
(**a**) Predicted cis-elements in the promoter regions of *AcHMA* genes. Motif names were shown nearby with different colors. (**b**) Distribution of cis-regulatory elements of *AcHMA* gene members

To better understand the complex regulatory networks that control gene expression in *AcHMA* genes, we identified and analyzed cis-elements in the promotor region on 2000 bp upstream of the starting site (Fig. 4a). The promoter regions of *AcHMA* genes contained the most stress-related cis-acting elements, which indicates their importance in stress-related pathways. This suggests that the genes may have a role in determining the plant’s reaction to external stimuli. Thus, understanding the role of these genes may provide insights into how plants adapt to their environments. Furthermore, we found that ABA (ABRE and CGTCA) and salicylic acid (SA) related cis-elements were detected in promoter regions of *AcHMA* genes associated with abiotic stresses (high temperature, wounds, and drought). These elements were also related to hormones (salicylic acid, gibberellin, MeJAE, Auxin, and ethylene) (Fig. 4b).

The heatmap is divided into four sections (growth, phytohormone regulation, light-responsive, and stress), in which rows represent the *AcHMA* genes and columns represent the cis-regulatory elements. The bar graph and the color from yellow to dark red represent the number of elements in each row and column.

### Differential expression patterns of AcHMA genes in various tissues

**Fig. 5:**
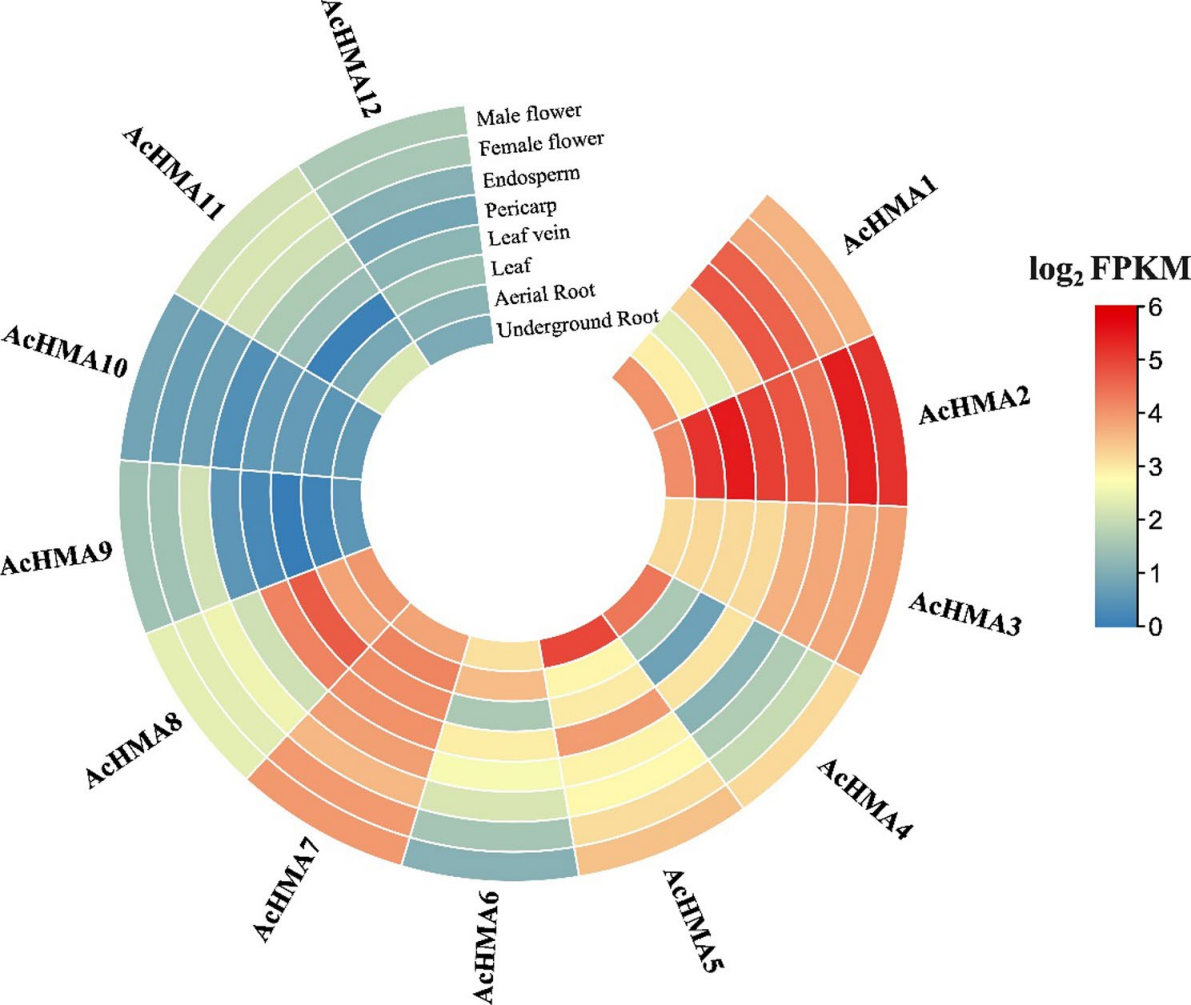
The expression heatmap of the 12 *AcHMA* genes in eight different tissues of the *A. catechu*

The transcriptomic data of *A. catechu* were used to calculate the gene FPKM expression values of *AcHMA* genes in different tissues (i.e., male and female flowers, endosperm, pericarp, leaf, and leaf vein, aerial and underground roots) (Table S6). The expression profiling reveals that *AcHMA5, AcHMA8, AcHMA3, AcHMA2*, and *AcHMA1* were highly expressed in root while *AcHMA1, AcHMA2*, and *AcHMA8* were upregulated in leaves and veins. Expression levels of *AcHMA1*, *AcHMA6*, *AcHMA7*, and *AcHMA8* were higher in male and female parts of the plant. Furthermore, *AcHMA1, AcHMA7*, and *AcHMA8* genes were highly expressed in the endosperm and pericarp. Notably, *AcHMA9, AcHMA10, AcHMA11*, and *AcHMA12* were low expressed in all tissues of *Areca* (Fig. 5; Table S6).

### Abiotic stress-induced expression patterns of AcHMA genes

**Fig. 6:**
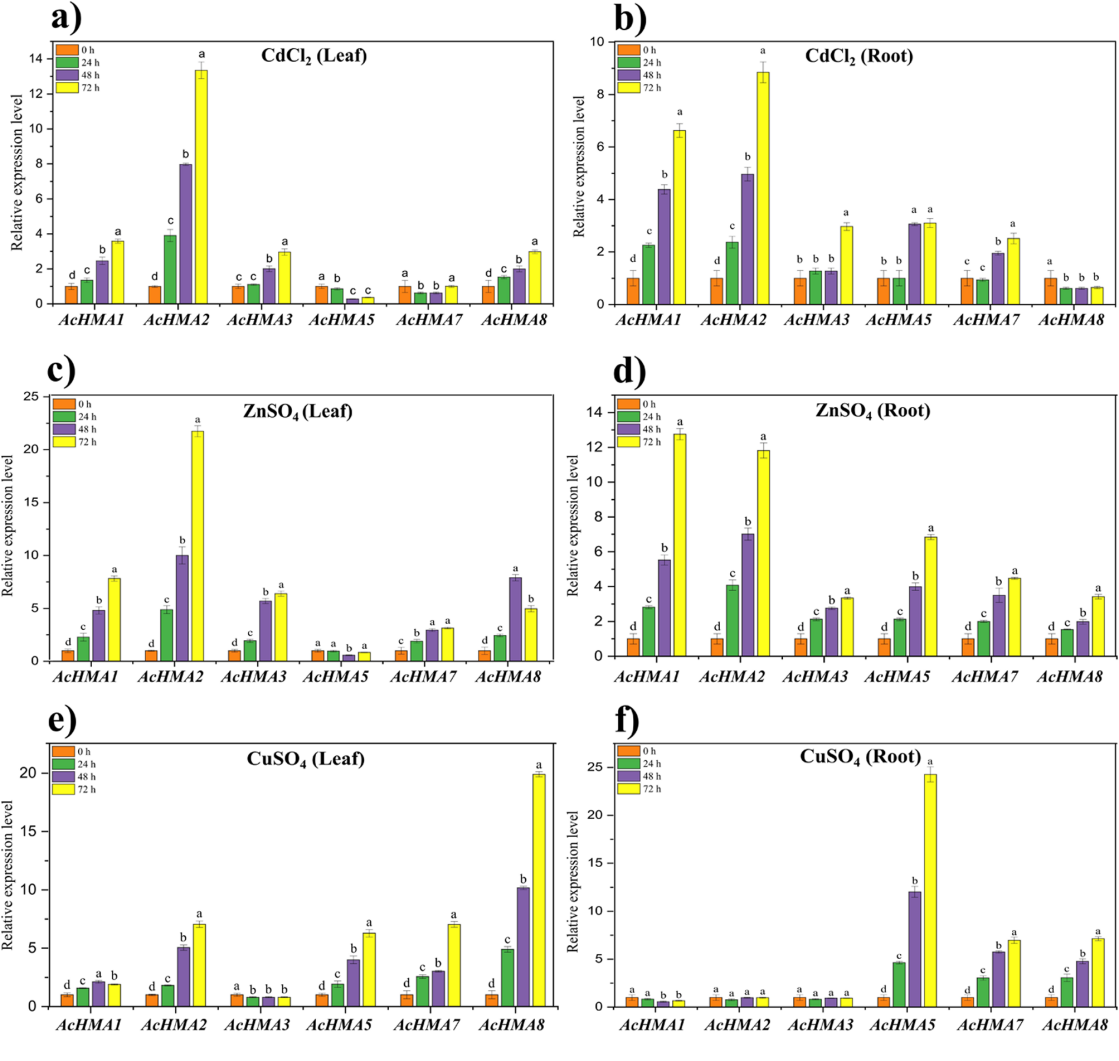
The expression pattern of *HMA* genes in *A. catechu* leaves and roots under heavy metals. *HMA* genes expression in *A. catechu* under (**a**) CdCl_2_ (leaf), (**b**) root; (**c**) ZnSO_4_ (leaf), (**d**) root, (**e**) CuSO_4_ (leaf), (**f** ) root at different time intervals (0 (C.K.), 24, 48, and 72 h). The data is presented in mean and standard error, *n* = 3, with asterisks indicating statistically significant differences (*p* ≤ 0.05) according to the LSD test

To reveal the response of *AcHMA* genes to heavy metals (Cd2+, Cu2 + and Zn2+) in roots and leaves, we detected the expression profile of *AcHMA* genes after heavy metal treatment. In response to Cd^2+^ treatment, *AcHMA1* and *AcHMA2* were highly upregulated genes; *AcHMA3* and *AcHMA8* exhibited moderate upregulation, while *AcHMA5* was a downregulated gene in leaves after 72 h. Similarly, all genes were highly expressed in roots under Cd^2+^ treatment except *AcHMA8* after 72 h (Fig. 6a, b). Furthermore, in response to Zn^2+^ treatment, in addition to *AcHMA5*, the expression levels of *AcHMA1*, *AcHMA2*, *AcHMA3* and *AcHMA*7 in leaves increased gradually with the extension of treatment time (Fig. 6c). In particular, in the root, all AcHMA genes’ expression levels increased significantly after treatment (Fig. 6d). In response to Cu^2+^ treatment, *AcHMA1*, *AcHMA2*, *AcHMA5, AcHMA7* and *AcHMA8* were upregulated in leaves, while *AcHMA3* downregulated in leaves (Fig. 6e). The gene expression levels of *AcHMA5*, *AcHMA7*, and *AcHMA8* in roots substantially rose as the treatment period increased (Fig. 6f).

## Discussion

Plants require a control system for managing metal homeostasis in order to balance the delivery of essential micronutrients (such as zinc, copper, and iron) to various organs while simultaneously preventing non-essential metals such as lead and cadmium from accumulating to toxic concentrations that may adversely affect the plant [35]. Heavy metal ATPase are transmembrane transporters that play a significant role in heavy metal transportation, distribution, detoxification, and accumulation in plants [36]. Previous studies have identified the HMA gene family in various plants, including 8 in *A. thaliana* [37], 9 in rice (*Oryza sativa L*.) [38], 11 in maize (*Zea mays L*.) [21], 11 in sorghum (*Sorghum bicolor L*.) [21], 17 in *Populus trichocarpa* [39], and 20 in soybean (*Glycine max L*.) [40], 31 in *Brassica napus* (*B. napus*) [41], 8 mulberry (*Morus*) [25], 21 barley *(Hordeum vulgare L.)* [42], 14 turnip (*Brassica rapa*) [43], 48 *tomato* (*Solanum lycopersicum*) [34], and 27 in *wheat (T. aestivum)* [22]. However, the heavy metal ATPase gene family within the *Arecaceae* family remains unreported in existing literature.

In this study, we identified 12 *AcHMA* genes in *Areca*. The number of *AcHMA* genes in *A.catechu* genome was lower than in tomato and wheat [22, 34] while almost closer to sorghum, rice and maize [21], suggesting a potential shared evolutionary or functional aspect in the context of *HMA* genes among these plant species. The *HMA* family members has been divided into several clades in different plant species based on substrate specificity and phylogenetic clustering. HMA proteins in *Arabidopsis* and potato were categorized into 6 clades [44], 5 in barley [42], 8 in flax [45], 7 in cotton [46], 11 in dragon fruit [47], and 3 in maize [48]. The *AcHMA* gene family in *A.catechu* was categorized into two sub-families (Cu/Ag P1B-ATPase and Zn/Co/Cd/Pb P1B-ATPase) and five sub-subfamilies (I-V) based on substrate specificity and phylogenetic clustering among *A. catechu*, *Arabidopsis*, *O. sative*, and *C.nucifera*. Our investigation into the *HMA* gene family in *A. catechu* aligns with the established pattern, suggesting a conserved organizational structure within the *HMA* gene family across plant species.

The similar structural pattern of *HMA* genes shows that they might have similar functions and connections in different plants, especially regarding their specific substances [39]. This helps us to understand the various roles these gene family play in different plants and how they adapt to their environments. It has been reported that *HMA* transporter activities are linked to the subcellular distribution of the *HMA* transporters. The *AtHMA1*, *OsHMA1, AtHMA6*, and *AtHMA8* identified both in *Arabidopsis* and rice, exhibited a chloroplast envelop localization, and were involved in Zinc translocation into the chloroplast [23, 49]. Similarly, *AtHMA2, OsHMA2, AtHMA4, AtHMA5, OsHMA5* and *OsHAM9* are located in plasma membrane confers tolerance to Zn/Cd/Cu [50–52]. The *OsHMA3* and *OsHMA4* in *Oryza sativa* are located at the tonoplast and help in cadmium and copper accumulation in vacule [50, 53].

Furthermore, P1B-ATPases have metal binding domains (MBDs) that act as cell transport regulators [54]. The distinctive cysteine structure (CxxC) in the HMA domain enables these genes to bind metal ions through thiol groups [55, 56]. P1B-ATPases, a type of transporter, are effective in expelling essential and toxic metal ions out of cells, ensuring a balance of metals (homeostasis) within the cellular environment. This mechanism is vital for maintaining the overall health and functionality of the cell [14, 57]. Understanding the exon-intron structure is crucial in evolution and comprehending the gene family. Significant evolutionary changes occur in eukaryotic genes due to intron gain or loss, which entails inserting and deleting gene segments [58]. Analyzing the exon-intron structure of *AcHMA* genes reveals conservation within the gene family despite divergent exon numbers. Although, the exon/intron count varies even within the same sub-family (Cu/Ag and Zn/Co/Cd/Pb P1B-ATPase). Exon/intron ratio variations within the gene family hint at functional diversification or evolutionary changes [59].

Cis-regulatory elements play a vital role in mediating stress responses, emphasizing their significance in the regulatory network [60]. Cis- regulatory elements and plant hormones, including ethylene (ET), salicylic acid (SA), abscisic acid (ABA), and jasmonic acid (JA), act as crucial signalling molecules that empower plants to adapt to abiotic stresses [60, 61]. The analysis of cis-regulatory elements of *AcHMA* family genes indicates the presence of multiple abiotic stress-responsive cis-elements. Specifically, elements like MYB, Myc, and STRE, abundant in the promoter region, exhibit a robust response to heavy metal stress, implying a potential role of *AcHMA* in the plant’s reaction to Cd and other heavy metal stress [62]. Understanding cis-acting elements in gene promoters, like G-box and ABRE, is crucial for unravelling plant stress responses. The abundance of cis-elements suggests that these genes may be triggered in response to various environmental stresses, potentially improving its overall stress tolerance [63].

According to previous studies, *HMAs* are thought to play a crucial role in detoxifying heavy metals in dicots by inhibiting the efflux of these metals into the cytosol through efflux mechanisms [14]. To understand the role of *AcHMAs* in *A.catechu*, we performed RNA-seq data analysis and qPCR to investigate the expression of *AcHMA* under normal conditions and heavy metal stress. In normal conditions, the *AcHMA1*-*AcHMA8* genes had high expression levels throughout many tissues. However, the expression pattern of these genes was altered in response to abiotic stimuli, thereby highlighting the diverse function of *AcHMA* genes. The diverse expression pattern of *AcHMA* genes suggests that they were widely involved in abiotic stress as well as the growth and development of *A.catechu*. As a result of heavy metals exposure, both roots and leaves exhibited high levels of *AcHMA* gene expression at 48 and 72 h. Notably, *AcHMA1* and *AcHMA2* genes exhibited the highest upregulation in response to Cd^2+^ and Zn^2+^ stresses in roots and leaves, while *AcHMA5* and *AcHMA8* showed significant upregulation in the presence of Cu^2+^.The higher expression level of *AcHMA1* and *AcHMA2* against Cd^2+^ stress indicates their role in cadmium transportation in roots and leaves. Previously, it was revealed that *AtHMA3* in *Arabidopsis*, *OsHMA3* in rice and *ZmHMA2* and *ZmHMA3* from maize has shown cadmium transport capabilities into root vacuoles of plants [1, 26, 30]. Based on these results, it appears that *HMA2* and *HMA3* genes play a similar role in regulating cadmium transport in diverse plant species, and this supports our conclusion regarding *AcHMA1* and *AcHMA2*. These results provide evidence that genes closely related to each other in plant phylogeny play a similar role in regulating cadmium transport to vacuolar sequestration, as well as subsequent root-to-shoot translocation in various plant species.

**Fig. 7:**
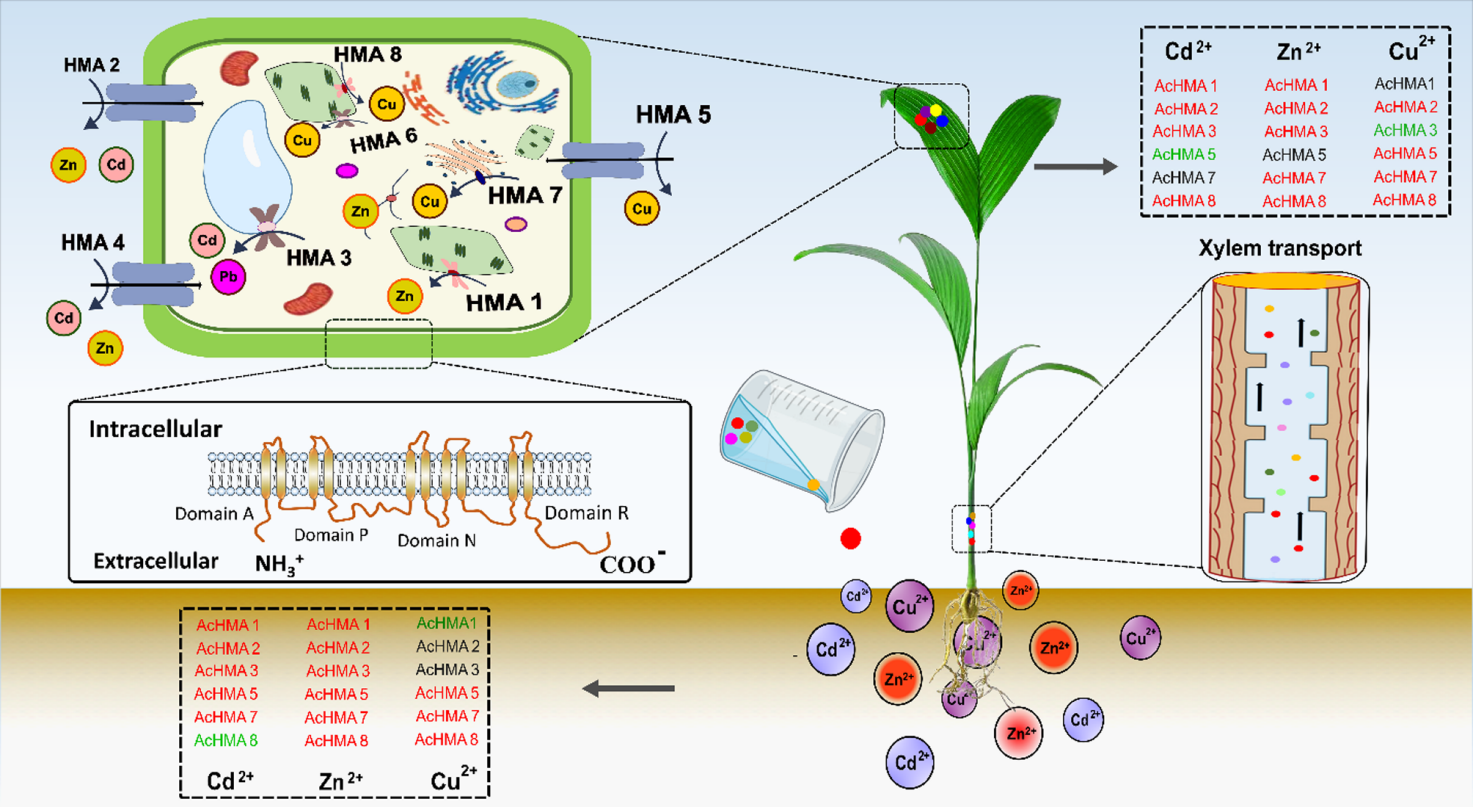
Schematic model showing subcellular distribution of *HMA* and expression of *AcHMA* transporters in roots and leaves of *A. catechu* under Cd^2+^, Zn^2+^ and Cu²^+^ stress. The red font shows a significant overexpression, the green font indicates significant downregulation, and the black font indicates no significant gene expression change

Similarly, as a result of Zn^2+^ treatment, our study revealed a notable upregulation of *AcHMA1, AcHMA2, AcHMA3, AcHMA7*, and *AcHMA8* both in leaves as well as in roots, suggesting their involvement in zinc transportation in the tonoplast. Similar gene expression patterns have been identified in both *O.sativa* and *A.thalian*, and our findings align with those earlier studies. As a result of zinc stress, previous studies reported elevated levels of *AtHMA1*, *OsHMA1*, and *OsHMA2* expression in the tonoplast, indicating that these genes play a role in zinc transport [14, 23, 27]. In response to Cu²^+^ stress, most of the *AcHMAs* show differential expression patterns. Notably, *AcHMA5* and *AcHMA8* were upregulated in roots and leaves, suggesting their potential roles in Cu transport or detoxification. Additionally, *AcHMA2*, *AcHMA5*, and *AcHMA7* exhibited moderate expression in leaves, while *AcHMA1*, *AcHMA2*, and *AcHMA3* appeared downregulated in roots. Our findings are consistent with those of the existing literature. Noteworthy, *AcHMA5* upregulation in roots corresponds to the reported involvement of *AtHMA5, OsHMA5*, and *PtHMA5* in Cu transport from the roots to the shoots and Cu detoxification. Furthermore, the increased expression of *AcHMA8* in leaves reflects the recognized roles of *AtHMA8*, *PtHMA7*, and *PtHMA8* in Cu transport, emphasizing how these roles were conserved across different plant species throughout evolution. In conclusion, based on the gene expression profiles under different heavy metal stress treatments, we proposed a schematic model, which summarized the key *AcHMA* genes that play role in response to different heavy metal stress in *Areca* (Fig. 7). This study comprehensively explores the functional roles and expression patterns within the HMA gene family of *A. catechu.* Our results will lay a foundation for further analysis of how *AcHMA* gene responds to heavy metal stress in areca.

### Electronic supplementary material

Below is the link to the electronic supplementary material.

Supplementary Material 1

## Data Availability

The Illumina Hiseq 4000 RNA-seq data was deposited at NCBI (accession number: PRJNA767949). All data generated or analyzed during this study can be found in Additional files.
